# Associations of childhood exposure to interparental physical violence and verbal conflict with the risk of adult-onset diabetes

**DOI:** 10.7189/jogh.16.04161

**Published:** 2026-05-15

**Authors:** Qi Gao, Licong Su, Yaping Yu, Lunlun Wu, Zhou Ma, Weirong Ren, Yan Cui, Yuxin Lin, Fan Luo, Ruqi Xu, Hongxue Yu, Junwu Dong, Yanqin Li, Honglan Wei

**Affiliations:** 1 Department of Nephrology, Wuhan Fourth Hospital, Wuhan Clinical Research Centre for Metabolic Chronic Kidney Disease, Wuhan, Hubei, China; 2 Division of Nephrology, National Clinical Research Centre for Kidney Disease, State Key Laboratory of Organ Failure Research, Nanfang Hospital, Southern Medical University, Guangzhou, China.; 3 School of Medicine, Jianghan University, Wuhan, Hubei, China; 4 Division of Nephrology, People’s Hospital of Yangjiang Affiliated to Guangdong Medical University, Yangjiang, China; 5 Division of Nephrology, Huashan Hospital, Fudan University, Shanghai, China

## Abstract

**Background:**

Adverse childhood experiences have been associated with health across the life course. However, the associations of childhood exposure to interparental physical violence and verbal conflict with the risk of adult-onset diabetes remain unclear.

**Methods:**

A total of 6769 participants without diabetes were enrolled. We assessed childhood exposure to interparental physical violence and verbal conflict through the standard questionnaire. The outcome was adult-onset diabetes, defined as the new occurrence of diabetes during follow-up. Diabetes was ascertained if any of the following criteria were met: self-reported physician diagnosis, current use of glucose-lowering medication, glycated haemoglobin A1c ≥6.5%, fasting plasma glucose ≥126 mg/dL, or two-hour postprandial plasma glucose ≥200 mg/dL. The associations between childhood exposure to interparental violence and adult-onset diabetes were assessed using Cox proportional hazards models.

**Results:**

The mean age was 58.6 years, and 3146 participants (46.5%) were male. Among all participants, 565 (8.3%) reported childhood exposure to interparental physical violence, and 1523 (22.5%) reported exposure to interparental verbal conflict. During a follow-up of seven years, 854 participants (12.6%) developed diabetes. The multivariable Cox model showed that childhood exposure to interparental physical violence and verbal conflict was associated with a 67.0% and 20.0% increased risk of adult-onset diabetes, respectively (for physical violence adjusted hazard ratio (aHR) = 1.67; 95% confidence interval (CI) = 1.36–2.05; for verbal conflict aHR = 1.20; 95% CI = 1.02–1.40).

**Conclusions:**

Childhood exposure to interparental physical violence and verbal conflict was associated with a higher risk of adult-onset diabetes. These findings underscore the long-term health implications of early adverse family environments on offspring.

Adverse childhood experiences (ACEs) have increasingly been recognised as important social determinants of health and are closely associated with health across the life course [1]. ACEs commonly include household dysfunction (such as violence at home, parental substance abuse, and parental separation), violence (such as dating violence or direct victimisation), childhood maltreatment (psychological, physical, or sexual abuse), and other adversities such as peer bullying and economic hardship [2]. The prevalence of ACEs has become a growing global concern and varies according to nativity, race/ethnicity, and socioeconomic status [2–5]. A national USA survey reported that in 2009, 59% of adults recalled at least one adverse event [3], and this proportion rose to 63.9% a decade later [6]. A population-based cohort study of 1 097 628 children in Denmark found that nearly half experienced some degree of adversity between 1980 and 2025 [7]. Moreover, evidence from the China Health and Retirement Longitudinal Study (CHARLS) revealed that more than 90% of respondents reported at least one ACE [8].

Beyond developmental and psychological consequences in childhood, ACEs substantially increase the risk of subsequent mental health disorders, including depression, anxiety, and posttraumatic stress disorder [9–14]. In addition, the literature has demonstrated associations between ACEs and adverse cardiometabolic outcomes, such as myocardial infarction, stroke, coronary heart disease, and hypertension [3,8,15–18], as well as obesity and diabetes [19–23].

Interparental physical violence and verbal conflict are common manifestations of household violence, a core component of ACEs. These forms of interparental aggression represent not only direct spousal abuse but also an indirect form of violence toward children, who are exposed to a hostile family environment even if not physically targeted. In the USA, 14–18% of adults reported witnessing interparental physical violence during childhood [24,25]. Another study, including 14 256 women aged 15–49 years, found that 43.8% had witnessed violence in their home of origin during childhood [26]. Repeated exposure to such violence may carry significant implications for long-term health and well-being [17].

Nevertheless, the majority of existing studies have investigated ACEs as a whole or concentrated predominantly on childhood maltreatment. Prospective evidence specifically addressing childhood exposure to interparental physical violence and verbal conflict and their long-term health consequences remains limited [17]. To address this gap in knowledge, we aimed to explore the association between childhood exposure to interparental violence and the risk of adult-onset diabetes in later life.

## METHODS

### Setting, data source, and population

For this study, we derived data from the CHARLS database. The baseline survey was initiated in 2011 and recruited participants aged ≥45 years through a multistage probability sampling strategy that covered 28 provinces, 150 counties/districts, and 450 villages/urban communities across China [27]. Follow-up surveys have been conducted every two to three years (including 2013, 2015, and 2018 waves), collecting detailed information on demographics, socioeconomic status, health behaviours, physical and psychological health, and biomarkers. Data were collected through face-to-face personal interviews, supplemented by physical measurements and blood sample collection. We conducted the study in accordance with the ethical principles of the Declaration of Helsinki and reported the results in accordance with the STROBE guidelines. In addition, we adhered to the GRABDROP guidelines (Table S1 in the **Online Supplementary Document**) [28,29].

In this study, we initially included 16 825 Chinese middle-aged and older adults aged ≥45 years from the 2011 wave (Figure S1 in the **Online Supplementary Document**). After linkage with the 2014 early-life survey, 13 837 participants were matched. We excluded participants with missing data on interparental physical violence or verbal conflict (n = 1862), the parental education data (n = 194), childhood bullying experience data (n = 34), childhood sibling violence data (n = 69), childhood hunger data (n = 55), or without any follow-up wave (n = 42). We further excluded those with missing information on glycated haemoglobin A1c (HbA1c), or blood glucose (n = 3549), and individuals with diabetes at baseline (n = 1254). Finally, we selected 6769 participants for the final analysis.

### Definition of childhood interparental physical violence and verbal conflict

We obtained information on childhood exposure to interparental physical violence and verbal conflict from the 2014 wave of the CHARLS life history survey questionnaire. Participants were asked to retrospectively report their experiences before the age of 17 years through three questions: ‘Did your parents often quarrel?’ ‘Has your father ever beaten up your mother?’ and ‘Has your mother ever beaten up your father?’ Response options included often, sometimes, not very often, and never. Childhood exposure to witnessing interparental verbal conflict was defined as responding often or sometimes to question one. Similarly, childhood exposure to witnessing interparental physical violence was defined as responding often or sometimes to either question two or question three [17,30].

### Determination of adult-onset diabetes

The outcome of interest was adult-onset diabetes, defined as the new occurrence of diabetes during follow-up among participants who were free of diabetes at baseline (2011 wave). Diabetes was ascertained if any of the following criteria were met: self-reported physician diagnosis, current use of glucose-lowering medication, HbA1c ≥6.5%, fasting plasma glucose ≥126 mg/dL, or two-hour postprandial plasma glucose ≥200 mg/dL. Specifically, self-reported diagnosis was based on a positive response to the question: ‘Have you been diagnosed with diabetes or high blood glucose by a doctor?’ Current medication use was identified if participants answered ‘yes’ to any of the following options: taking Chinese traditional medicine, taking Western modern medicine, or using insulin injections in response to the question ‘Are you now taking any of the following treatments to treat or control your diabetes?’ It should be noted that blood biomarker data were only available in the 2011 and 2015 waves; therefore, diabetes diagnoses in the 2013 and 2018 waves were determined solely from self-reported physician diagnosis and current medication use. Participants were followed from baseline until the first occurrence of diabetes, or the last available survey wave, whichever came first.

### Assessment of covariates

We obtained baseline data from standardised questionnaires, laboratory measurements, and physical examinations conducted during the 2011 wave. Covariates included age, sex (male or female), education levels (middle school or below, and high school or above), residence (rural or urban), marital status (married or unmarried), smoking (yes or no), drinking (yes or no), body mass index, systolic blood pressure, diastolic blood pressure, cardiovascular disease (CVD), hypertension, HbA1c, and blood glucose. In addition, several variables, including highest parental education (illiterate, middle school or below, or high school or above), childhood hunger (yes or no), frequency of childhood bullying (never, rarely, sometimes, or frequently), and frequency of childhood sibling violence (never, rarely, sometimes, or frequently), were obtained from the 2014 early-life survey (Methods S1 in the **Online Supplementary Document**).

### Statistical analysis

We presented baseline characteristics as means and standard deviations (SDs), medians, interquartile ranges (IQRs), or frequencies (percentages), as appropriate. We assessed baseline comparability using standardised mean differences (SMDs). Given that the risk of diabetes strongly depends on age, Kaplan-Meier curves stratified by childhood exposure to interparental physical violence and verbal conflict were plotted using attained age at baseline and at the end of follow-up to better reflect cumulative incidence [17,18,31]. Associations between childhood exposure to interparental physical violence, verbal conflict, and adult-onset diabetes were evaluated using Cox proportional hazards models with follow-up time (time since baseline) as the underlying time scale. We tested proportional hazards assumptions using the ‘cox.zph’ function in *R* (R Core Team, Vienna, Austria). Results were expressed as hazard ratios (HRs) with 95% confidence intervals (CIs). We used a directed acyclic graph to visualise the selection of covariates (Figure S2 in the **Online Supplementary Document**). In multivariable analyses, model 1 was adjusted for age and sex. Model 2 further adjusted for smoking, drinking, education, rural residence, highest parental education, childhood hunger, childhood bullying experience, and childhood sibling violence.

### Subgroup analysis

We examined potential effect modifications in the associations between interparental physical violence, verbal conflict, and adult-onset diabetes across several predefined subgroups. We conducted stratifications by age (<60 *vs.* ≥60 years), sex (male *vs.* female), rural residence (yes *vs.* no), smoking (yes *vs.* no), drinking (yes *vs.* no), childhood hunger (yes *vs.* no), childhood bullying experience (never to rarely *vs.* sometimes to frequently), and childhood sibling violence (never to rarely *vs.* sometimes to frequently). Effect modification was assessed by adding an interaction term to each multivariable Cox regression model. All subgroup analyses were prespecified and interpreted cautiously, given the potential for limited statistical power.

### Sensitivity analysis

Considering the difficulty in precisely capturing the onset time of diabetes, we applied interval-censored Cox regression [32]. To further reduce baseline imbalances, we constructed one-on-one propensity score-matched cohorts for both interparental physical violence (unexposed *vs.* exposed) and interparental verbal conflict (unexposed *vs.* exposed) [33]. We estimated propensity scores (PSs) using multivariable logistic regression models adjusted for covariates listed in model 2, and matching was performed by nearest-neighbour matching without replacement within a calliper of 0.05 × SD of the PS. The SMD<0.1 indicated well-balanced baseline characteristics. We further applied two PSs weighting approaches to verify the stability of our results [34]. The inverse probability of treatment weighting (IPTW) method assigned weights of 1/(1 – PS) for the unexposed group and 1/PS for the exposed group, where PS was derived as described above. The overlap weighting (OW) method assigned weights of PS for the unexposed group and 1-PS for the exposed group. Considering a substantial number of participants with missing data were excluded from the original cohort, potential selection bias may have been introduced. We applied IPTW and OW to address this issue. PS representing the probability of being included in the final analytical data set were estimated using multivariable logistic regression, with age, sex, smoking, drinking, and rural residence as covariates. For IPTW, participants included in the final analysis were weighted by 1/PS. For OW, weights were defined as 1 – PS for included participants. The estimated weights for included participants were then incorporated into the Cox proportional hazards models to evaluate the association between the exposures and adult-onset diabetes. We repeated the analyses using a modified exposure definition, in which ‘never’ was classified as unexposed and ‘rarely,’ ‘sometimes,’ or ‘frequently’ were classified as exposed. Given that the early-life survey was conducted in 2014, we further restricted the follow-up period to post-2014 to examine the robustness of our findings. We further redefined the outcome as self-reported diabetes only.

### Additional analysis

We further performed an E-value analysis to assess the potential impact of unmeasured confounding that would be required to fully null the observed associations of interparental physical violence and verbal conflict with adult-onset diabetes [35]. We conducted all statistical analyses using *R*, version 4.5.1. A two-sided *P*-value <0.05 was considered statistically significant. Data analyses were performed between 16 April 2025 and 12 August 2025.

## RESULTS

### Baseline characteristics of the study population

Among 15 743 CHARLS participants aged ≥45 years, we included 6769 individuals in the final analysis. The mean age was 58.6 years (SD = 8.8), and 3146 (45.8%) participants were male. Among all participants, 565 (8.3%) reported childhood exposure to interparental physical violence, and 1523 (22.5%) reported exposure to interparental verbal conflict (**Table 1**). Compared with participants who never experienced interparental physical violence and verbal conflict, those who were frequently exposed were more likely to be female and to have experienced childhood hunger, childhood bullying, and childhood sibling violence (Tables S2 and S3 in the **Online Supplementary Document**).

**Table 1:** Baseline characteristics of the study population stratified by frequency of witnessing interparental physical violence and verbal conflict*

Variable	Overall (n = 6769)	Frequency of witnessing interparental physical violence				SMD	Frequency of witnessing interparental verbal conflict				SMD
		**Never (n = 5345)**	**Rarely (n = 859)**	**Sometimes (n = 440)**	**Frequently (n = 125)**		**Never (n = 3255)**	**Rarely (n = 1991)**	**Sometimes (n = 1175)**	**Frequently (n = 348)**	
Age in years, x̄ (SD)	58.6 (8.8)	58.6 (8.9)	58.2 (8.8)	58.4 (8.6)	59.1 (8.9)	0.052	59.6 (9.0)	57.5 (8.6)	57.7 (8.7)	58.34 (8.5)	0.131
Male	3146 (46.5)	2431 (45.5)	461 (53.7)	208 (47.3)	46 (36.8)	0.177	1430 (43.9)	1025 (51.5)	551 (46.9)	140 (40.2)	0.123
Education						0.072					0.134
*Middle school or below*	6111 (90.3)	4799 (89.8)	789 (91.9)	406 (92.3)	117 (93.6)		2984 (91.7)	1764 (88.6)	1034 (88.0)	329 (94.5)	
*High school or above*	658 (9.7)	546 (10.2)	70 (8.1)	34 (7.7)	8 (6.4)		271 (8.3)	227 (11.4)	141 (12.0)	19 (5.5)	
Rural residence	4501 (66.5)	3515 (65.8)	598 (69.6)	305 (69.3)	83 (66.4)	0.052	2239 (68.8)	1271 (63.8)	762 (64.9)	229 (65.8)	0.056
Childhood hunger	4864 (71.9)	3748 (70.1)	671 (78.1)	341 (77.5)	104 (83.2)	0.159	2266 (69.6)	1438 (72.2)	882 (75.1)	278 (79.9)	0.130
Frequency of childhood bullying experience†						0.347					0.365
*Never*	4819 (71.2)	3988 (74.6)	491 (57.2)	276 (62.7)	64 (51.2)		2612 (80.2)	1287 (64.6)	710 (60.4)	210 (60.3)	
*Rarely*	981 (14.5)	691 (12.9)	200 (23.3)	66 (15.0)	24 (19.2)		317 (9.7)	412 (20.7)	194 (16.5)	58 (16.7)	
*Sometimes*	696 (10.3)	489 (9.1)	123 (14.3)	66 (15.0)	18 (14.4)		217 (6.7)	220 (11.0)	220 (18.7)	39 (11.2)	
*Frequently*	273 (4.0)	177 (3.3)	45 (5.2)	32 (7.3)	19 (15.2)		109 (3.3)	72 (3.6)	51 (4.3)	41 (11.8)	
Frequency of childhood sibling violence						0.308					0.266
*Never*	5784 (85.4)	4710 (88.1)	650 (75.7)	329 (74.8)	95 (76.0)		2977 (91.5)	1608 (80.8)	918 (78.1)	281 (80.7)	
*Rarely*	607 (9.0)	394 (7.4)	153 (17.8)	44 (10.0)	16 (12.8)		163 (5.0)	271 (13.6)	137 (11.7)	36 (10.3)	
*Sometimes*	312 (4.6)	202 (3.8)	49 (5.7)	52 (11.8)	9 (7.2)		94 (2.9)	99 (5.0)	100 (8.5)	19 (5.5)	
*Frequently*	66 (1.0)	39 (0.7)	7 (0.8)	15 (3.4)	5 (4.0)		21 (0.6)	13 (0.7)	20 (1.7)	12 (3.4)	
Highest parental education						0.067					0.090
*Illiterate*	3984 (58.9)	3134 (58.6)	522 (60.8)	253 (57.5)	75 (60.0)		2016 (61.9)	1088 (54.6)	677 (57.6)	203 (58.3)	
*Middle school or below*	2574 (38.0)	2031 (38.0)	322 (37.5)	174 (39.5)	47 (37.6)		1139 (35.0)	837 (42.0)	461 (39.2)	137 (39.4)	
*High school or above*	211 (3.1)	180 (3.4)	15 (1.7)	13 (3.0)	3 (2.4)		100 (3.1)	66 (3.3)	37 (3.1)	8 (2.3)	
Married	6057 (89.5)	4785 (89.5)	764 (88.9)	397 (90.2)	111 (88.8)	0.043	2898 (89.0)	1803 (90.6)	1062 (90.4)	294 (84.5)	0.100
Smoking	2017 (29.8)	1561 (29.2)	280 (32.6)	152 (34.5)	24 (19.2)	0.188	956 (29.4)	620 (31.1)	349 (29.7)	92 (26.4)	0.053
Drinking	2300 (34.0)	1750 (32.7)	337 (39.2)	170 (38.6)	43 (34.4)	0.082	1010 (31.0)	744 (37.4)	425 (36.2)	121 (34.8)	0.072
BMI in kg/m^2^, MD (IQR)	23.0 (20.8–25.6)	23.1 (20.9–25.6)	22.7 (20.7–25.2)	23.2 (20.9–25.9)	22.9 (20.8–25.7)	0.056	23.0 (20.8–25.5)	23.2 (20.9–25.5)	23.1 (20.9–25.9)	22.6 (20.6–25.5)	0.028
SBP in mmHg, MD (IQR)	126.0 (114.0–141.0)	127.0 (115.0–142.0)	124.0 (112.0–138.0)	126.0 (112.0–141.0)	126.0 (115.0–138.8)	0.080	128.0 (115.0–143.0)	125.0 (113.0–140.0)	126.0 (113.0–140.00)	125.0 (112.0–139.0)	0.069
DBP in mmHg, MD (IQR)	75.0 (67.0–84.0)	75.0 (68.0–84.0)	74.0 (66.0–83.0)	74.0 (67.0–83.0)	74.0 (67.0–84.0)	0.075	75.0 (68.0–84.0)	74.0 (67.0–83.0)	75.0 (67.0–84.0)	74.5 (66.0–85.0)	0.040
Cardiovascular disease	997 (14.7)	776 (14.5)	127 (14.8)	72 (16.4)	22 (17.6)	0.049	484 (14.9)	273 (13.7)	170 (14.5)	70 (20.1)	0.088
Hypertension	2643 (39.0)	2137 (40.0)	292 (34.0)	172 (39.1)	42 (33.6)	0.152	1369 (42.1)	725 (36.4)	424 (36.1)	125 (35.9)	0.064
HbA1c in %, MD (IQR)	5.1 (4.9–5.4)	5.1 (4.9–5.3)	5.1 (4.9–5.3)	5.1 (4.9–5.4)	5.1 (4.9–5.4)	0.063	5.1 (4.9–5.4)	5.1 (4.9–5.3)	5.1 (4.9–5.3)	5.1 (4.9–5.4)	0.048
Blood glucose in mg/dL, MD (IQR)	100.6 (93.4–108.0)	100.6 (93.4–107.8)	100.6 (93.3–108.7)	100.3 (92.7–108.8)	99.2 (92.3–108.5)	0.039	100.6 (93.4–108.3)	100.4 (93.6–107.5)	100.4 (93.1–108.0)	99.9 (92.3–109.3)	0.025

BMI – body mass index, DBP – diastolic blood pressure, HbA1c – glycated haemoglobin A1c, IQR – interquartile range, MD – median, SD – standard deviation, SMD – standardised mean difference, SBP – systolic blood pressure, x̄ – mean

*Presented as n (%) unless specified otherwise.

†Childhood bullying experience was defined as a bullying experience by peers in a neighbourhood or school before the participants were 17 y of age.

### Childhood violence experience and adult-onset diabetes

**Figure 1:**
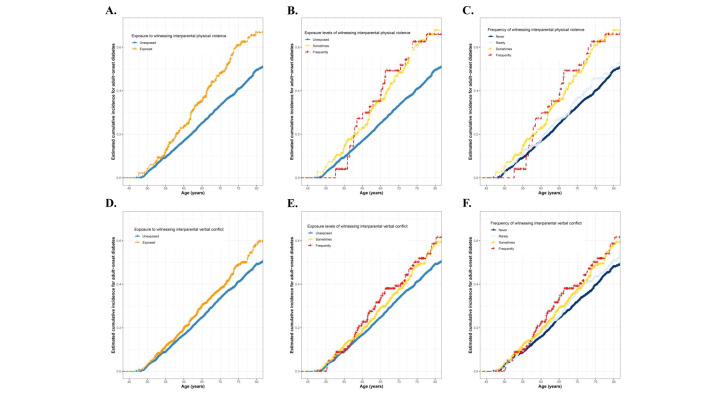
Cumulative incidence of adult-onset diabetes according to interparental physical violence and verbal conflict experience. **Panel A.** Exposure to witnessing interparental physical violence. **Panel B.** Exposure levels of witnessing interparental physical violence. **Panel C.** Frequency of witnessing interparental physical violence. **Panel D.** Exposure to witnessing interparental verbal conflict. **Panel E.** Exposure levels of witnessing interparental verbal conflict. **Panel F.** Frequency of witnessing interparental verbal conflict. *Unexposed was defined as witnessing interparental physical violence/verbal conflict never or rarely when participants were younger than 17 years. †Exposed was defined as witnessing interparental physical violence/verbal conflict sometimes or frequently when participants were younger than 17 years.

During a follow-up of 7.0 years (IQR = 6.9–7.0), a total of 43 044.5 person-years were accumulated, and 854 participants (12.6%) developed diabetes. Individuals exposed to interparental physical violence or verbal conflict in childhood had a higher cumulative incidence of diabetes than those without such experiences (for physical violence 31.5 *vs.* 18.8 per 1000 person-years; for verbal conflict 22.9 *vs.* 19.0 per 1000 person-years). The highest incidence rates were observed among those frequently exposed to violence (for physical violence 35.1 per 1000 person-years; for verbal conflict 26.7 per 1000 person-years) (**Figure 1**, **Table 2**). Proportional hazards assumptions were satisfied, and after adjusting for confounding factors included in model 2, childhood exposure to interparental physical violence (adjusted hazard ratio (aHR) = 1.67; 95% CI = 1.36–2.05) and verbal conflict (aHR = 1.20; 95% CI = 1.02–1.40) were associated with a 67.0% and 20.0% increased risk of adult-onset diabetes, respectively. Analysis by frequency of exposure revealed that participants with more frequent exposure might have a higher risk of diabetes. For physical violence, with an aHR of 1.08 (95% CI = 0.88–1.32) for rarely, with an aHR of 1.65 (95% CI = 1.31–2.08) for sometimes, and for frequently with an aHR of 1.84 (95% CI = 1.25–2.72). For verbal conflict, the corresponding aHRs were 1.07 (95% CI = 0.91–1.26), 1.18 (95% CI = 0.98–1.43), and 1.38 (95% CI = 1.04–1.82), respectively (Table S4 in the **Online Supplementary Document**).

**Table 2:** Associations of childhood exposure to interparental physical violence and verbal conflict with the risk of adult-onset diabetes

	Total, n	Events (incidence rate), n (%)*	Crude model		Model 1†		Model 2†	
**HR (95% CI)**	***P*-value**	**HR (95% CI)**	***P*-value**	**HR (95% CI)**	***P*-value**
**Interparental physical violence**								
Exposure to witnessing interparental physical violence								
*Unexposed‡*	6204	744 (18.8)	Ref.		Ref.		Ref.	
*Exposed§*	565	110 (31.5)	1.70 (1.39–2.07)	<0.001	1.70 (1.39–2.07)	<0.001	1.67 (1.36–2.05)	<0.001
Exposure levels of witnessing interparental physical violence								
*Unexposed‡*	6204	744 (18.8)	Ref.		Ref.		Ref.	
*Sometimes*	440	83 (30.5)	1.63 (1.30–2.05)	<0.001	1.65 (1.31–2.07)	<0.001	1.63 (1.30–2.05)	<0.001
*Frequently*	125	27 (35.1)	1.92 (1.31–2.82)	0.001	1.87 (1.27–2.75)	0.001	1.82 (1.23–2.68)	0.002
Frequency of witnessing interparental physical violence								
*Never*	5345	632 (18.5)	Ref.		Ref.		Ref.	
*Rarely*	859	112 (20.5)	1.08 (0.89–1.33)	0.426	1.10 (0.90–1.35)	0.33	1.08 (0.88–1.32)	0.484
*Sometimes*	440	83 (30.5)	1.65 (1.32–2.08)	<0.001	1.67 (1.33–2.10)	<0.001	1.65 (1.31–2.08)	<0.001
*Frequently*	125	27 (35.1)	1.95 (1.32–2.86)	0.001	1.90 (1.29–2.79)	0.001	1.84 (1.25–2.72)	0.002
**Interparental verbal conflict**								
Exposure to witnessing interparental verbal conflict								
*Unexposed‡*	5246	634 (19.0)	Ref.		Ref.		Ref.	
*Exposed§*	1523	220 (22.9)	1.20 (1.03–1.40)	0.019	1.21 (1.04–1.41)	0.014	1.20 (1.02–1.40)	0.025
Exposure levels of witnessing interparental verbal conflict								
*Unexposed‡*	5246	634 (19.0)	Ref.		Ref.		Ref.	
*Sometimes*	1175	162 (21.8)	1.18 (1.00–1.41)	0.055	1.19 (1.00–1.41)	0.053	1.16 (0.97–1.38)	0.101
*Frequently*	348	58 (26.7)	1.42 (1.08–1.85)	0.011	1.39 (1.06–1.82)	0.016	1.36 (1.04–1.79)	0.025
Frequency of witnessing interparental physical violence								
*Never*	3255	387 (18.7)	Ref.		Ref.		Ref.	
*Rarely*	1991	247 (19.4)	1.04 (0.88–1.22)	0.659	1.08 (0.92–1.27)	0.338	1.07 (0.91–1.26)	0.395
*Sometimes*	1175	162 (21.8)	1.16 (0.97–1.40)	0.106	1.20 (1.00–1.45)	0.050	1.18 (0.98–1.43)	0.078
*Frequently*	348	58 (26.7)	1.40 (1.06–1.85)	0.016	1.41 (1.07–1.86)	0.015	1.38 (1.04–1.82)	0.025

CI – confidence interval, HR – hazard ratio, ref – reference

*The incidence rate was expressed per 1000 person-years.

†Model 1 was adjusted for sex and age. Model 2 included model 1 and further adjustments for smoking, drinking, education, rural residence, highest parental education, childhood hunger, childhood bullying experience, and childhood sibling violence

‡Unexposed was defined as witnessing interparental physical violence/verbal conflict never or rarely when participants were <17 y.

§Exposed was defined as witnessing interparental physical violence/verbal conflict sometimes or frequently when participants were <17 y.

In joint-effect analyses, 5128 individuals (75.8%) were unexposed to both types of childhood violence, whereas 447 individuals (6.6%) were exposed to both types. Regardless of interparental verbal conflict status, exposure to interparental physical violence was consistently associated with higher diabetes risk (without verbal conflict aHR = 2.05, 95% CI = 1.39–3.03; with verbal conflict aHR = 1.61 (95% CI = 1.27–2.03). (**Table 3**). Interestingly, the diabetes risk appeared higher among those unexposed to verbal conflict than among those exposed, but the difference did not reach statistical significance (*P* = 0.271). However, this finding should be interpreted cautiously, given the limited sample size.

**Table 3:** Joint associations of childhood exposure to interparental physical violence and verbal conflict with the risk of adult-onset diabetes*

Exposure to witnessing interparental physical violence	Exposure to witnessing interparental verbal conflict, HR (95% CI)	
	**Unexposed (n = 5246)**†	**Exposed (n = 1523)**‡
Unexposed (n = 6204)†	Ref.	1.08 (0.89–1.30)
Exposed (n = 565)‡	2.05 (1.39–3.03)§	1.61 (1.27–2.03)§

CI – confidence interval, HR – hazard ratio, ref – reference

*The numbers of total participants and incident cases for each joint exposure category are as follows: unexposed to both (n = 5128, events = 607); exposed to verbal conflict only (n = 1076, events = 137); exposed to physical violence only (n = 118, events = 27); and exposed to both physical violence and verbal conflict (n = 447, events = 83).

†Unexposed was defined as witnessing interparental physical violence/verbal conflict never or rarely when participants were <17 y.

‡Exposed was defined as witnessing interparental physical violence/verbal conflict sometimes or frequently when participants were <17 y.

§No significant difference was observed between the two HRs (Wald χ^2^ = 1.21, *P*-value = 0.271).

### Subgroup analysis

**Figure 2:**
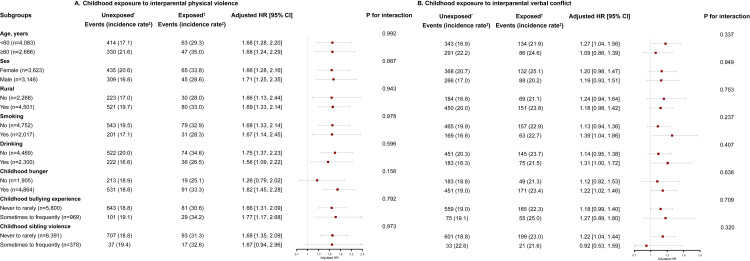
Subgroup analyses by potential modifiers of the associations between childhood exposure to interparental violence and the risk of adult-onset diabetes. **Panel A.** Exposure to witnessing interparental physical violence. **Panel B.** Exposure to witnessing interparental verbal conflict. The model was adjusted for age, sex, smoking, drinking, education, rural residence, highest parental education, childhood hunger, childhood bullying experience, and childhood sibling violence. *Unexposed was defined as witnessing interparental physical violence/verbal conflict never or rarely when participants were younger than 17 years. †Exposed was defined as witnessing interparental physical violence/verbal conflict sometimes or frequently when participants were younger than 17 years. ‡The incidence rate was expressed per 1000 person-years. CI – confidence interval, HR – hazard ratio, ref – reference.

None of the stratified variables, including age, sex, rural residence, smoking, drinking, childhood hunger, childhood bullying experience, and childhood sibling violence, significantly modified the associations between childhood exposure to interparental physical violence or verbal conflict and diabetes risk (all *P* > 0.05) (**Figure 2**).

### Sensitivity analysis and additional analysis

The results were similar to the primary. First, similar associations were observed when applying interval-censoring Cox regression models (for physical violence aHR = 1.67, 95% CI = 1.46–1.88; for verbal conflict aHR = 1.25, 95% CI = 1.09–1.42). Second, after conducting one-on-one propensity score matching, 986 and 2798 pairs of participants exposed to interparental physical violence and verbal conflict were matched, with baseline covariates well balanced between the exposed and unexposed groups (SMD<0.1). In the matched cohorts, the associations were slightly attenuated (for physical violence aHR = 1.42, 95% CI = 1.02–1.97; for verbal conflict aHR = 1.19, 95% CI = 0.97–1.45). Furthermore, both IPTW and OW Cox regression models confirmed that exposure to interparental physical violence and verbal conflict was associated with an increased risk of diabetes. After applying IPTW and OW weighting to the final included population, the results remained consistent with the main findings. After modifying the definitions of the exposure and outcome, the results remained consistent with the primary findings (Tables S5–12 in the **Online Supplementary Document**). The calculated E-values were 2.73 for interparental physical violence and 1.69 for verbal conflict.

## DISCUSSION

In this large, nationally representative, prospective cohort study of 6769 Chinese middle-aged and older adults with a median follow-up of seven years, 565 (8.3%) and 1523 (22.5%) participants reported childhood exposure to interparental physical violence and verbal conflict, respectively. We observed a positive association between witnessing these forms of adverse childhood household violence and the risk of adult-onset diabetes. These associations were consistent across multiple subgroups and sensitivity analyses. Our findings indicated that the quality of parental relationships during childhood might be an important determinant of long-term health outcomes in offspring.

ACEs have a profound impact on childhood morbidity and mortality and were increasingly recognised as preventable adverse social determinants of health [2,36]. Traditionally, interventions to prevent cardiovascular-kidney-metabolic diseases have focused primarily on modifying health behaviours in adults. However, the latest American Heart Association statements emphasised that childhood is a critical period for identifying and mitigating risk across the life course, recommending that social determinants of health screening be conducted annually for all children [37]. Developing prevention and management strategies during childhood that were effective across diverse settings remained a crucial public health priority.

Previous studies have mainly focused on the accumulation of ACEs or exposure to multiple ACEs simultaneously, and their associations with diverse outcomes, including depression, CVD, diabetes, and chronic kidney disease [8,13,38–45]. Some studies reported a continuous dose-response relationship between the number of ACEs and the risk of mental health disorders and CVD [8,40,42]. However, other research has suggested a threshold effect. For example, Ma *et al.* found that exposure to fewer than four ACEs was not significantly associated with mental health disorders (odds ratio (OR) = 1.21; 95% CI = 0.71–2.05), whereas exposure to four or more ACEs showed a positive linear association with risk (OR = 2.03; 95% CI = 1.20–3.44) [13]. When focusing on specific types of ACEs, prior research has predominantly examined childhood maltreatment, while studies on household dysfunction, particularly interparental violence, and its long-term consequences for offspring, have remained limited. A study from the CHARLS, including 10 961 participants, did not find a significant association between adverse interparental relationships and mental health disorders (OR = 1.36; 95% CI = 0.88–2.11). Wu *et al.* reported that exposure to witnessing interparental physical violence was associated with a 1.26-fold increased risk of adult-onset total CVD, and that the risk increased with higher frequency of witnessing interparental physical violence [17]. Similarly, in our study, after adjusting for health behaviours, the association between childhood exposure to interparental physical violence and verbal conflict and adult-onset diabetes remained significant, with risk increasing as exposure frequency rose. These findings suggested that behavioural factors alone may not fully explain the elevated risk.

The specific mechanisms linking early-life exposure to violence with the development of chronic diseases in adulthood remained unclear. Previous studies have proposed that early psychological stress may become biologically embedded through long-term alterations in immune, endocrine, and metabolic pathways [46]. Epigenetic modifications could promote chronic inflammation, while hormonal dysregulation and maladaptive behaviours might further amplify disease risk. Over time, this persistent proinflammatory state accelerated biological pathways that contributed to ageing-related conditions, including CVD, diabetes, autoimmune disorders, and premature mortality [46,47]. More recently, Consortium *et al.* employed two-step and multivariable Mendelian randomisation analyses to explore potential mediating roles of inflammatory, metabolic, and neuroendocrine biomarkers in the association between childhood maltreatment and adult multimorbidity [48]. These analyses indicated that up to 11% of the effect of childhood maltreatment on multimorbidity was mediated by triglycerides, 8% by HbA1c, and up to 7% by high-density lipoprotein cholesterol, whereas no significant mediation was observed for inflammatory proteins or cortisol. However, these mechanisms were not directly assessed in the present study, and further research incorporating biological markers is needed to clarify the underlying pathways.

The major strength of this study lies in the use of a large, nationally representative cohort, which enabled both qualitative and quantitative assessment of the association between childhood exposure to interparental physical violence and verbal conflict and the risk of adult-onset diabetes. Nevertheless, several limitations should be acknowledged. First, exposure to interparental violence in childhood was assessed retrospectively through questionnaires, which might be subject to recall bias. Second, the specific subtypes of diabetes could not be determined in this study, which limited further exploration of the relationships between interparental violence and diabetes subtypes. In addition, the lack of precise information on the timing of diabetes onset might introduce bias; however, our reanalysis using an interval-censoring Cox regression model yielded results consistent with the main findings. Third, the exact timing of violence exposure during childhood was not available, which further limited us in examining how exposures at different developmental stages might differentially influence later health outcomes. Fourth, as with all observational studies, residual confounding was inevitable. The calculated E-values provided some indication of the robustness of the observed associations to unmeasured confounding; however, given that some values were modest, the findings should be interpreted cautiously. Finally, the study population consisted of middle-aged and older Chinese adults, and the generalizability of our findings to other populations warrants further investigation.

## CONCLUSIONS

Childhood exposure to interparental physical violence and verbal conflict was associated with a higher risk of developing adult-onset diabetes. These findings highlight the potential long-term health implications of early adverse family environments on offspring. Future studies, including longitudinal and intervention-based research, are warranted to better understand the underlying mechanisms and assess whether addressing early-life adversities could improve metabolic health later in life.

## Additional material


Online Supplementary Document

## References

[1] Shonkoff JP Garner AS The lifelong effects of early childhood adversity and toxic stress. Pediatrics. 2012;129:e232–46. 10.1542/peds.2011-2663 22201156

[2] Suglia SF Koenen KC Boynton-Jarrett R Chan PS Clark CJ Danese A Childhood and Adolescent Adversity and Cardiometabolic Outcomes: A Scientific Statement From the American Heart Association. Circulation. 2018;137:e15–28. 10.1161/CIR.0000000000000536 29254928 PMC7792566

[3] Centers for Disease Control and Prevention (CDC) Adverse childhood experiences reported by adults – five states, 2009. MMWR Morb Mortal Wkly Rep. 2010;59:1609–13. 21160456

[4] Cronholm PF Forke CM Wade R Bair-Merritt MH Davis M Harkins-Schwarz M Adverse Childhood Experiences: Expanding the Concept of Adversity. Am J Prev Med. 2015;49:354–61. 10.1016/j.amepre.2015.02.001 26296440

[5] Slopen N Shonkoff JP Albert MA Yoshikawa H Jacobs A Stoltz R Racial Disparities in Child Adversity in the U.S.: Interactions With Family Immigration History and Income. Am J Prev Med. 2016;50:47–56. 10.1016/j.amepre.2015.06.013 26342634

[6] Swedo EA Aslam MV Dahlberg LL Niolon PH Guinn AS Simon TR Prevalence of Adverse Childhood Experiences Among U.S. Adults - Behavioral Risk Factor Surveillance System, 2011-2020. MMWR Morb Mortal Wkly Rep. 2023;72:707–15. 10.15585/mmwr.mm7226a2 37384554 PMC10328489

[7] Rod NH Bengtsson J Budtz-Jørgensen E Clipet-Jensen C Taylor-Robinson D Andersen AN Trajectories of childhood adversity and mortality in early adulthood: a population-based cohort study. Lancet. 2020;396:489–97. 10.1016/S0140-6736(20)30621-8 32798491

[8] Han Y Rong J Chen J Lv Y Jing F The impact of adverse childhood experiences on cardiovascular disease risk in middle-aged and older adults. Soc Sci Med. 2025;383:118446. 10.1016/j.socscimed.2025.118446 40716341

[9] Thurston C Murray AL Franchino-Olsen H Silima M Hemady CL Meinck F Prospective Longitudinal Associations Between Adverse Childhood Experiences and Adult Mental Health Outcomes: Systematic Review and Meta-Analysis. Trauma Violence Abuse. 2025:15248380251358223. 10.1177/15248380251358223 40819337 PMC13578656

[10] Byansi W Galvin M Chiwaye L Luvuno Z Kim AW Sundararajan R Adverse childhood experiences, traumatic events, and mental health among adults at two outpatient psychiatric facilities in Johannesburg, South Africa: a cross-sectional analysis. BMC Psychiatry. 2023;23:581. 10.1186/s12888-023-05085-0 37563695 PMC10413614

[11] Guo Y Wu Y Fan S Wang H Prevalence of Adverse Childhood Experiences and Long-Term Associations With Mental Health Among Adults: A Nationwide Cross-Sectional Study in China. MedComm (2020). 2025;6:e70366. 10.1002/mco2.70366 40904705 PMC12401931

[12] Mildred-Short G Tashjian SM Adverse childhood experiences and adult psychopathology: A latent class analysis approach. Child Abuse Negl. 2025;169:107672. 10.1016/j.chiabu.2025.107672 40925198

[13] Ma N Ji X Shi Y Wang Q Wu J Cui X Adverse childhood experiences and mental health disorder in China: A nationwide study from CHARLS. J Affect Disord. 2024;355:22–30. 10.1016/j.jad.2024.03.110 38548193

[14] Yu P Wang X Liu J Luo H Yi Y Adverse childhood experiences, marital status and depressive symptoms in later life among the Chinese middle-aged and older adults: the mediating role of marital status. BMC Public Health. 2024;24:2246. 10.1186/s12889-024-19787-x 39160540 PMC11331659

[15] Su S Jimenez MP Roberts CT Loucks EB The role of adverse childhood experiences in cardiovascular disease risk: a review with emphasis on plausible mechanisms. Curr Cardiol Rep. 2015;17:88. 10.1007/s11886-015-0645-1 26289252 PMC4941633

[16] Norman RE Byambaa M De R Butchart A Scott J Vos T The long-term health consequences of child physical abuse, emotional abuse, and neglect: a systematic review and meta-analysis. PLoS Med. 2012;9:e1001349. 10.1371/journal.pmed.1001349 23209385 PMC3507962

[17] Cui C Liu L Li H Qi Y Song J Han N Childhood Exposure to Interparental Physical Violence and Adult Cardiovascular Disease. JAMA Netw Open. 2024;7:e2451806. 10.1001/jamanetworkopen.2024.51806 39705033 PMC11662254

[18] Cui C Liu L Guo Z Song J Zheng Y Zhang Z Childhood Exposure to Multiple Types of Violence and Adult Cardiovascular Disease. Eur J Prev Cardiol. 2025:zwaf292. 10.1093/eurjpc/zwaf292 40390600

[19] Midei AJ Matthews KA Interpersonal violence in childhood as a risk factor for obesity: a systematic review of the literature and proposed pathways. Obes Rev. 2011;12:e159–72. 10.1111/j.1467-789X.2010.00823.x 21401850 PMC3104728

[20] Basu A McLaughlin KA Misra S Koenen KC Childhood Maltreatment and Health Impact: The Examples of Cardiovascular Disease and Type 2 Diabetes Mellitus in Adults. Clin Psychol (New York). 2017;24:125–39. 28867878 10.1111/cpsp.12191 PMC5578408

[21] Elsenburg LK van Wijk KJE Liefbroer AC Smidt N Accumulation of adverse childhood events and overweight in children: A systematic review and meta-analysis. Obesity (Silver Spring). 2017;25:820–32. 10.1002/oby.21797 28371524

[22] Huffhines L Noser A Patton SR The Link Between Adverse Childhood Experiences and Diabetes. Curr Diab Rep. 2016;16:54. 10.1007/s11892-016-0740-8 27112958 PMC5292871

[23] Rich-Edwards JW Spiegelman D Lividoti Hibert EN Jun HJ Todd TJ Kawachi I Abuse in childhood and adolescence as a predictor of type 2 diabetes in adult women. Am J Prev Med. 2010;39:529–36. 10.1016/j.amepre.2010.09.007 21084073 PMC3003936

[24] Thompson RS Bonomi AE Anderson M Reid RJ Dimer JA Carrell D Intimate partner violence: prevalence, types, and chronicity in adult women. Am J Prev Med. 2006;30:447–57. 10.1016/j.amepre.2006.01.016 16704937

[25] Bensley L Van Eenwyk J Wynkoop Simmons K Childhood family violence history and women’s risk for intimate partner violence and poor health. Am J Prev Med. 2003;25:38–44. 10.1016/S0749-3797(03)00094-1 12818308

[26] Bazo-Alvarez JC Copez-Lonzoy A Ipanaqué-Zapata M Bazalar-Palacios J Rivera EL Flores-Ramos EC Witnessing inter-parental violence in childhood and help-seeking behaviours in violence against women in Peru. BMC Public Health. 2024;24:1022. 10.1186/s12889-024-18467-0 38609932 PMC11015581

[27] Zhao Y Hu Y Smith JP Strauss J Yang G Cohort profile: the China Health and Retirement Longitudinal Study (CHARLS). Int J Epidemiol. 2014;43:61–8. 10.1093/ije/dys203 23243115 PMC3937970

[28] von Elm E Altman DG Egger M Pocock SJ Gøtzsche PC Vandenbroucke JP The Strengthening the Reporting of Observational Studies in Epidemiology (STROBE) statement: guidelines for reporting observational studies. Ann Intern Med. 2007;147:573–7. 10.7326/0003-4819-147-8-200710160-00010 17938396

[29] Rudan I Song P Adeloye D Campbell H Journal of Global Health’s Guidelines for Reporting Analyses of Big Data Repositories Open to the Public (GRABDROP): preventing ‘paper mills’, duplicate publications, misuse of statistical inference, and inappropriate use of artificial intelligence. J Glob Health. 2025;15:01004. 10.7189/jogh.15.01004 40587200 PMC12208284

[30] Lin L Cao B Chen W Li J Zhang Y Guo VY Association of Adverse Childhood Experiences and Social Isolation With Later-Life Cognitive Function Among Adults in China. JAMA Netw Open. 2022;5:e2241714. 10.1001/jamanetworkopen.2022.41714 36367722 PMC9652754

[31] Thiébaut AC Bénichou J Choice of time-scale in Cox’s model analysis of epidemiologic cohort data: a simulation study. Stat Med. 2004;23:3803–20. 10.1002/sim.2098 15580597

[32] Kim DK Regression analysis of interval-censored survival data with covariates using log-linear models. Biometrics. 1997;53:1274–83. 10.2307/2533496 9423249

[33] Zhao QY Luo JC Su Y Zhang YJ Tu GW Luo Z Propensity score matching with R: conventional methods and new features. Ann Transl Med. 2021;9:812. 10.21037/atm-20-3998 34268425 PMC8246231

[34] Thomas LE Li F Pencina MJ Overlap Weighting: A Propensity Score Method That Mimics Attributes of a Randomized Clinical Trial. JAMA. 2020;323:2417–8. 10.1001/jama.2020.7819 32369102

[35] Haneuse S VanderWeele TJ Arterburn D Using the E-Value to Assess the Potential Effect of Unmeasured Confounding in Observational Studies. JAMA. 2019;321:602–3. 10.1001/jama.2018.21554 30676631

[36] Grummitt LR Kreski NT Kim SG Platt J Keyes KM McLaughlin KA Association of Childhood Adversity With Morbidity and Mortality in US Adults: A Systematic Review. JAMA Pediatr. 2021;175:1269–78. 10.1001/jamapediatrics.2021.2320 34605870 PMC9059254

[37] Ndumele CE Rangaswami J Chow SL Neeland IJ Tuttle KR Khan SS Cardiovascular-Kidney-Metabolic Health: A Presidential Advisory From the American Heart Association. Circulation. 2023;148:1606–35. 10.1161/CIR.0000000000001184 37807924

[38] Qiao Y Zhu D Zhao M Magnussen CG Xi B Adverse childhood experience, adopting a healthy lifestyle in adulthood, and risk of cardiovascular diseases. J Affect Disord. 2024;362:450–8. 10.1016/j.jad.2024.07.023 39009308

[39] Tung EL Wroblewski KE Makelarski JA Glasser NJ Lindau ST Childhood Parental Incarceration and Adult-Onset Hypertension and Cardiovascular Risk. JAMA Cardiol. 2023;8:927–35. 10.1001/jamacardio.2023.2672 37647038 PMC10469273

[40] Wang W Liu Y Yang Y Jiang W Ni Y Han X Adverse childhood and adulthood experiences and risk of new-onset cardiovascular disease with consideration of social support: a prospective cohort study. BMC Med. 2023;21:297. 10.1186/s12916-023-03015-1 37553602 PMC10408183

[41] Shen Z Li C Fang Y Chen H Song Y Cui J Association between adverse childhood experiences with chronic kidney diseases in middle-aged and older adults in mainland China. Sci Rep. 2025;15:6469. 10.1038/s41598-025-91232-4 39987259 PMC11847003

[42] Liu Y Wang C Liu Y Association between adverse childhood experiences and later-life cardiovascular diseases among middle-aged and older Chinese adults: The mediation effect of depressive symptoms. J Affect Disord. 2022;319:277–85. 10.1016/j.jad.2022.09.080 36162657

[43] Campbell JA Farmer GC Nguyen-Rodriguez S Walker R Egede L Relationship between individual categories of adverse childhood experience and diabetes in adulthood in a sample of US adults: Does it differ by gender? J Diabetes Complications. 2018;32:139–43. 10.1016/j.jdiacomp.2017.11.005 29217352 PMC5750098

[44] Felitti VJ Anda RF Nordenberg D Williamson DF Spitz AM Edwards V Relationship of childhood abuse and household dysfunction to many of the leading causes of death in adults. The Adverse Childhood Experiences (ACE) Study. Am J Prev Med. 1998;14:245–58. 10.1016/S0749-3797(98)00017-8 9635069

[45] Gilbert LK Breiding MJ Merrick MT Thompson WW Ford DC Dhingra SS Childhood adversity and adult chronic disease: an update from ten states and the District of Columbia, 2010. Am J Prev Med. 2015;48:345–9. 10.1016/j.amepre.2014.09.006 25300735

[46] Miller GE Chen E Parker KJ Psychological stress in childhood and susceptibility to the chronic diseases of aging: moving toward a model of behavioral and biological mechanisms. Psychol Bull. 2011;137:959–97. 10.1037/a0024768 21787044 PMC3202072

[47] McEwen BS Protective and damaging effects of stress mediators. N Engl J Med. 1998;338:171–9. 10.1056/NEJM199801153380307 9428819

[48] Baltramonaityte V Karhunen V Felix JF Penninx B Cecil CAM Fairchild G Biological pathways underlying the relationship between childhood maltreatment and Multimorbidity: A two-step, multivariable Mendelian randomisation study. Brain Behav Immun. 2025;126:59–69. 10.1016/j.bbi.2025.01.024 39900145

