# Supplementary material for: Associations of childhood exposure to interparental physical violence and verbal conflict with the risk of adult-onset diabetes
Source: J Glob Health. 2026 May 15;16:04161. doi: 10.7189/jogh.16.04161 (PMC13178053; doi:10.7189/jogh.16.04161)
Supplement: Online Supplementary Document [file jogh-16-04161-s001.pdf]

Supplement to: Gao Q, Su L, Yu Y, Wu L, Ma Z, Ren W, Cui Y, Lin Y, Luo F, Xu R, Yu H, Dong J, Li Y, Wei H. Associations of childhood exposure to interparental physical violence and verbal conflict with the risk of adult-onset diabetes. J Glob Health. 2026;16:04161.

Figure S1.

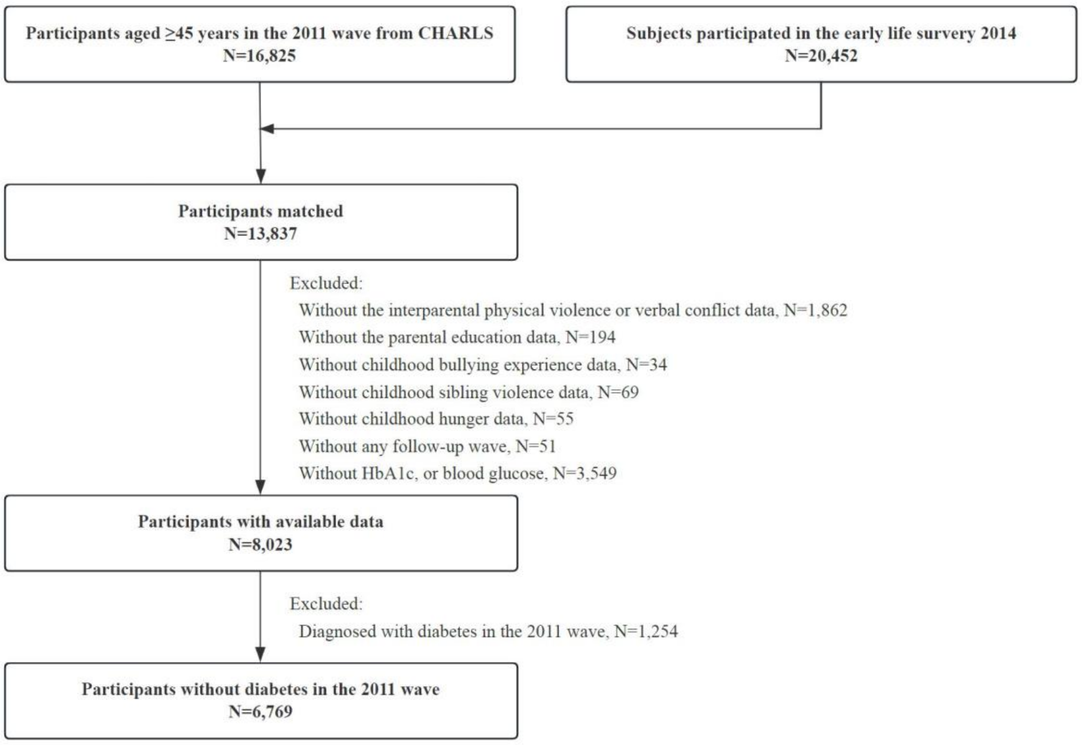

Figure s2.

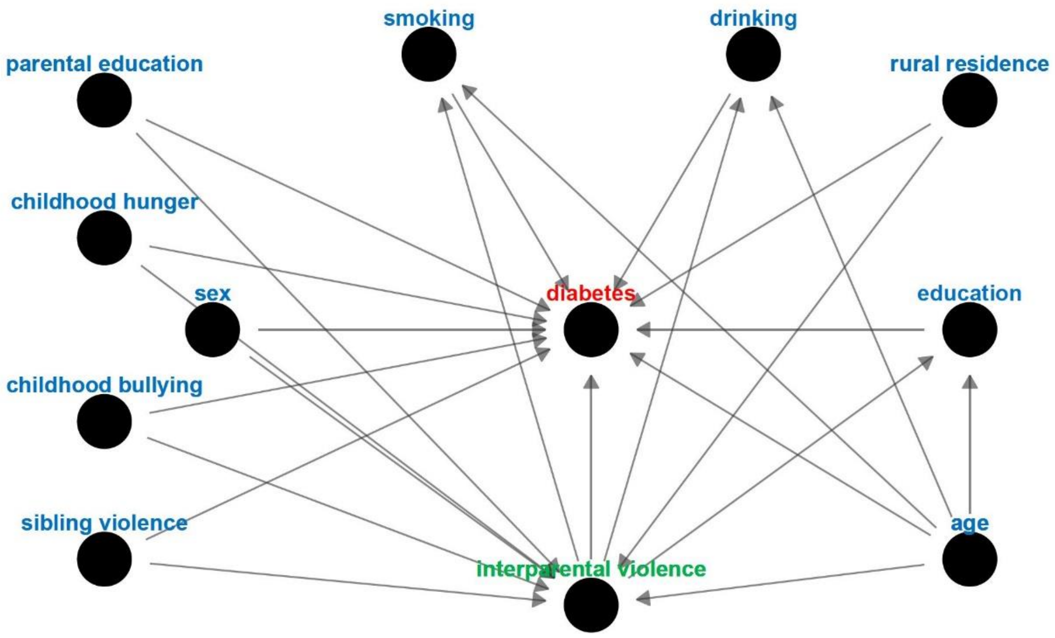

Method S1. Detailed definitions of covariates

Body mass index was calculated as weight in kilograms divided by the square of height in meters (kg/m²). Cardiovascular disease was defined based on a self-reported physician diagnosis of heart attack, coronary heart disease, angina, congestive heart failure, or other heart diseases. Hypertension

was defined if any of the following criteria were met: (1) self-reported physician diagnosis ("Have you been diagnosed with hypertension by a doctor?"), (2) current use of antihypertensive medication, (3) systolic blood pressure (SBP) $\geq$140 mmHg, or (4) diastolic blood pressure (DBP) $\geq$90 mmHg. SBP and DBP were calculated as the mean of three seated measurements. Highest parental education was defined as the highest educational attainment of either parent and was assessed using the question: "What is the highest level of education your mother/father completed?" Childhood hunger was determined using the question: "When you were a child before age 17, was there ever a time when your family did not have enough food to eat?" The frequency of childhood bullying was assessed using two questions: "When you were a child, how often were you picked on or bullied by kids in your neighborhood?" and "When you were a child, how often were you picked on or bullied by kids in your school?" The frequency of childhood sibling violence was assessed using the question: "When you were growing up, how often did your brother or sister ever hit you?" Response options for these items included often, sometimes, rarely, or never.

Table S1. Outline of JoGH guideline items

**JoGH guideline items**
1. Please list all papers published by each co-author in previous 3 years that were based on secondary analysis of a big data repository
1. **Gao Q**, Ren W, Du X, Yu Y, Ma Z, Cui Y, Song X, Wu Z, Zhang Z, Liao Q, Tian M, Wei H, Dong J. Sex-Specific Associations Between Depression and Cardiovascular-Kidney-Metabolic Syndrome Progression: A National Cohort Study. Eur J Prev Cardiol. 2026 Mar 9:zwag135. doi: 10.1093/eurjpc/zwag135.<br>2. Liang Z, **Su L**, Shi Y, Cai G, Wu W, An S, Zhang X. Triglyceride glucose-a body shape index (TyG-ABSI) as a potential indicator for cognitive impairment: Results from two nationally representative cohort studies. J Affect Disord. 2026 Mar 1;396:120809. doi: 10.1016/j.jad.2025.120809.<br>3. **Li Y**, **Gao Q**, **Luo F**, **Lin Y**, **Xu R**, Li P, Zhang Y, Liu J, Zhan H, **Su L**. L-shaped association of triglyceride glucose-body mass index and self-rated mental health among the middle-aged and older adults: a national cohort study in China. Front Public Health. 2025 Nov 25;13:1672881. doi: 10.3389/fpubh.2025.1672881<br>4. **Yu H**, **Gao Q**, **Lin Y**, **Luo F**, Li P, Zhang Y, Liu J, **Xu R**, **Li Y**, **Su L**. Effect of high normal body mass index and its trajectory on risk of new-onset hypertension among Chinese adults: a national prospective cohort study. Front Cardiovasc Med. 2025 Nov 21;12:1684124. doi: 10.3389/fcvm.2025.1684124.<br>5. Zhang X, Liang Z, **Su L**, Wang Z, Shi Y, Cai G, Deng Z, An S, Wu W. Metabolic phenotypes and pulmonary function in aging: the mediating roles of cognitive function and depression in CHARLS. BMC Public Health. 2025 Oct 31;25(1):3689. doi: 10.1186/s12889-025-24927-y.<br>6. **Gao Q**, **Luo F**, **Yu H**, **Lin Y**, **Xu R**, Li P, Zhang Y, Liu J, **Su L**, **Li Y**. U-shaped association between triglyceride-glucose index and all-cause mortality among critically ill pediatrics: a population-based retrospective cohort study. Cardiovasc Diabetol. 2024 Jun 26;23(1):222. doi: 10.1186/s12933-024-02310-2.<br>7. **Gao Q**, **Lin Y**, **Xu R**, **Luo F**, Chen R, Li P, Zhang Y, Liu J, Deng Z, **Li Y**, **Su L**, Nie S. Positive association of triglyceride-glucose index with new-onset hypertension among adults: a national cohort study in China. Cardiovasc Diabetol. 2023 Mar 16;22(1):58. doi: 10.1186/s12933-023-01795-7.
2. Please explain the key elements of your study design and the use of the available datasets that make your study an original scientific contribution
This study aims to explore the association of childhood exposure to interparental physical violence and verbal conflict with the risk of adult-onset diabetes.<br>Data were obtained from the China Health and Retirement Longitudinal Study (CHARLS), a nationally representative prospective cohort of middle-aged and older adults in China. CHARLS collected information on demographics, socioeconomic status, health behaviors, physical health, and biomarkers through repeated follow-up surveys. Importantly, the 2014 life-history survey retrospectively captured early-life experiences, allowing the linkage of childhood family environments with adult health outcomes.<br>This study yields the following findings: First, it identifies a significant association between childhood exposure to interparental physical violence and verbal conflict and adult-onset diabetes. Second, analysis by frequency of exposure revealed that participants with more frequent exposure might have a higher risk of diabetes. Finally, the findings are thoroughly validated across various subgroups and robust sensitivity analyses.
3. Please list all publications that addressed similar research questions in the same

dataset and indicate where you cited them in your paper

1. Han Y, Rong J, Chen J, Lv Y, Jing F. The impact of adverse childhood experiences on cardiovascular disease risk in middle-aged and older adults. Soc Sci Med. 2025;383:118446. doi:10.1016/j.socscimed.2025.118446 (Reference 3 in our manuscript)

2. Ma N, Ji X, Shi Y, et al. Adverse childhood experiences and mental health disorder in China: A nationwide study from CHARLS. *J Affect Disord*. 2024;355:22-30. doi:10.1016/j.jad.2024.03.110 (Reference 13 in our manuscript)

3. Cui C, Liu L, Li H, Qi Y, Song J, Han N, Wang Z, Shang X, Sheng C, Balmer L, Wu Z. Childhood Exposure to Interparental Physical Violence and Adult Cardiovascular Disease. JAMA Netw Open. 2024 Dec 2;7(12):e2451806. doi: 10.1001/jamanetworkopen.2024.51806. (Reference 17 in our manuscript)

4. Cui C, Liu L, Guo Z, et al. Childhood Exposure to Multiple Types of Violence and Adult Cardiovascular Disease. Eur J Prev Cardiol. Published online May 20, 2025. doi:10.1093/eurjpc/zwaf292 (Reference 18 in our manuscript)

4. Please explain how you addressed multiple testing through an appropriately rigorous statistical threshold and indicate this in the methods section

In this study, the independent variables are childhood exposure to interparental physical violence and verbal conflict, and the dependent variable is the occurrence of adult-onset diabetes. The covariates include demographic characteristics, childhood socioeconomic and contextual factors.

We used Cox proportional hazards models to investigate the associations of interparental violence and adult-onset diabetes. We additionally applied several subgroup analyses and sensitivity analyses including interval-censored Cox regression model, propensity score matching, inverse probability of treatment weighting, and overlap weighting.

We performed all analyses in R software, version 4.5.1. All statistical tests were two-sided, and statistical significance was defined as a P-value <0.05. To address potential concerns related to multiple comparisons in secondary analyses, results from subgroup and sensitivity analyses were interpreted cautiously.

5. Please declare to what extent have AI chatbots been used in developing your paper and to which parts of the paper did they contribute

No artificial intelligence chatbots were employed in the drafting of this manuscript.

Abbreviations: JoGH, journal of global health.

Table S2. Baseline characteristics of included and excluded participants

Variable	Overall (n=15,743)	Excluded (n=8,974)	Included (n=6,769)	SMD
Age, years	59.5 (9.8)	60.1 (10.4)	58.6 (8.8)	0.158
Male, n (%)	7,741 (49.2)	4,595 (51.2)	3,146 (46.5)	0.095
Education, n (%)				0.131
Middle school or below	13,840 (87.9)	7,729 (86.1)	6,111 (90.3)	
High school or above	1,899 (12.1)	1,241 (13.8)	658 (9.7)	
Missing	4 (0.0)	4 (0.0)	0 (0.0)	
Rural residence, n (%)	9,568 (60.8)	5,067 (56.5)	4,501 (66.5)	0.207
Childhood hunger, n (%)				0.984
No	3,631 (23.1)	1,726 (19.2)	1,905 (28.1)	
Yes	9,185 (58.3)	4,321 (48.2)	4,864 (71.9)	
Missing	2,927 (18.6)	2,927 (32.6)	0 (0.0)	
Frequency of childhood bullying experience, n (%)				1.038
Never	8,920 (56.7)	4,101 (45.7)	4,819 (71.2)	
Rarely	1,805 (11.5)	824 (9.2)	981 (14.5)	
Sometimes	1,307 (8.3)	611 (6.8)	696 (10.3)	
Frequently	579 (3.7)	306 (3.4)	273 (4.0)	
Missing	3,132 (19.9)	3,132 (34.9)	0 (0.0)	
Frequency of childhood sibling violence, n (%)				1.055
Never	10,610 (67.4)	4,826 (53.8)	5,784 (85.4)	
Rarely	1,186 (7.5)	579 (6.5)	607 (9.0)	
Sometimes	616 (3.9)	304 (3.4)	312 (4.6)	
Frequently	128 (0.8)	62 (0.7)	66 (1.0)	
Missing	3,203 (20.3)	3,203 (35.7)	0 (0.0)	
Highest parental education, n (%)				0.984
Illiterate	7,675 (48.8)	3,691 (41.1)	3,984 (58.9)	
Middle school or below	4,574 (29.1)	2,000 (22.3)	2,574 (38.0)	
High school or above	711 (4.5)	500 (5.6)	211 (3.1)	
Missing	2,783 (17.7)	2,783 (31.0)	0 (0.0)	
Married, n (%)	13,684 (86.9)	7,627 (85.0)	6,057 (89.5)	0.135
Smoking, n (%)	4,527 (28.8)	2,510 (28.0)	2,017 (29.8)	0.040
Drinking, n (%)	5,301 (33.7)	3,001 (33.4)	2,300 (34.0)	0.011
BMI, kg/m²	22.9 [20.7, 25.51]	22.9 [20.5, 25.5]	23.0 [20.8, 25.6]	0.001
SBP, mmHg	127.0 [115.0, 143.0]	128.0 [116.0, 144.0]	126.0 [114.0, 141.0]	0.096
DBP, mmHg	75.0 [68.0, 84.0]	76.0 [68.0, 84.0]	75.0 [67.0, 84.0]	0.043
Cardiovascular disease, n (%)	2,300 (14.6)	1,303 (14.5)	997 (14.7)	0.006
Hypertension, n (%)	6,039 (38.4)	3,396 (37.8)	2,643 (39.0)	0.025
HbA1c, %	5.1 [4.9, 5.4]	5.2 [4.9, 5.5]	5.1 [4.9, 5.4]	0.298
Blood glucose, mg/dL	101.9 [94.1, 111.8]	106.0 [96.1, 127.6]	100.6 [93.4, 108.0]	0.501

Data were expressed as mean (SD), median [25th percentile, 75th percentile] or as frequencies (percentages).

Covariates missingness: education (n=4), childhood hunger (n=2,927), childhood bullying experience (n=3,132), childhood sibling violence (n=3,203), highest parental education (n=2,783), BMI (n=3,522), SBP (n=3,400), and DBP (n=3,398).

Childhood bullying experience was defined as a bullied experience by peers in a neighbourhood or school before the participants were 17 years of age.

Abbreviations: SMD, standardized mean difference; BMI, body mass index; SBP, systolic blood pressure; DBP, diastolic blood pressure; HbA1c, glycated hemoglobin A1c.

Table S3. Baseline characteristics of study population stratified by adult-onset diabetes

Variable	Overall (n=6,769)	Adult-onset diabetes<br>No (n=5,915)	 Yes (n=854)	SMD
Age, years	58.6 (8.8)	58.5 (8.9)	59.3 (8.5)	0.090
Male, n (%)	3,146 (46.5)	2,792 (47.2)	354 (41.5)	0.116
Education, n (%)				0.061
Middle school or below	6,111 (90.3)	5,327 (90.1)	784 (91.8)	
High school or above	658 (9.7)	588 (9.9)	70 (8.2)	
Rural residence, n (%)	4,501 (66.5)	3,900 (65.9)	601 (70.4)	0.095
Childhood hunger, n (%)	4,864 (71.9)	4,242 (71.7)	622 (72.8)	0.025
Frequency of witnessing interparental physical violence, n (%)				0.177
Never	5,345 (79.0)	4,713 (79.7)	632 (74.0)	
Rarely	859 (12.7)	747 (12.6)	112 (13.1)	
Sometimes	440 (6.5)	357 (6.0)	83 (9.7)	
Frequently	125 (1.8)	98 (1.7)	27 (3.2)	
Frequency of witnessing interparental verbal conflict, n (%)				0.101
Never	3,255 (48.1)	2,868 (48.5)	387 (45.3)	
Rarely	1,991 (29.4)	1,744 (29.5)	247 (28.9)	
Sometimes	1,175 (17.4)	1,013 (17.1)	162 (19.0)	
Frequently	348 (5.1)	290 (4.9)	58 (6.8)	
Frequency of childhood bullying experience, n (%)				0.044
Never	4,819 (71.2)	4,219 (71.3)	600 (70.3)	
Rarely	981 (14.5)	857 (14.5)	124 (14.5)	
Sometimes	696 (10.3)	607 (10.3)	89 (10.4)	
Frequently	273 (4.0)	232 (3.9)	41 (4.8)	
Frequency of childhood sibling violence, n (%)				0.045
Never	5,784 (85.4)	5,064 (85.6)	720 (84.3)	
Rarely	607 (9.0)	527 (8.9)	80 (9.4)	
Sometimes	312 (4.6)	266 (4.5)	46 (5.4)	
Frequently	66 (1.0)	58 (1.0)	8 (0.9)	
Highest parental education, n (%)				0.064
Illiterate	3,984 (58.9)	3,462 (58.5)	522 (61.1)	
Middle school or below	2,574 (38.0)	2,263 (38.3)	311 (36.4)	
High school or above	211 (3.1)	190 (3.2)	21 (2.5)	
Married, n (%)	6,057 (89.5)	5,308 (89.7)	749 (87.7)	0.064
Smoking, n (%)	2,017 (29.8)	1,785 (30.2)	232 (27.2)	0.067
Drinking, n (%)	2,300 (34.0)	2,042 (34.5)	258 (30.2)	0.092
BMI, kg/m^2	23.0 [20.8, 25.6]	22.9 [20.7, 25.3]	24.5 [21.7, 27.1]	0.052
SBP, mmHg	126.0 [114.0, 141.0]	126.0 [114.0, 141.0]	130.0 [119.0, 144.0]	0.171
DBP, mmHg	75.0 [67.0, 84.0]	74.0 [67.0, 83.0]	77.0 [69.0, 86.0]	0.164
Cardiovascular disease, n (%)	997 (14.7)	818 (13.8)	179 (21.0)	0.189
Hypertension, n (%)	2,643 (39.0)	2,202 (37.2)	441 (51.6)	0.289
HbA1c, %	5.1 [4.9, 5.4]	5.1 [4.8, 5.3]	5.3 [5.0, 5.5]	0.496
Blood glucose, mg/dL	100.6 [93.4, 108.0]	99.9 [92.9, 107.1]	105.6 [97.2, 114.8]	0.405

Data were expressed as mean (SD), median [25th percentile, 75th percentile] or as frequencies (percentages).

Covariates missingness: BMI (n=922), SBP (n=898), and DBP (n=898).

Childhood bullying experience was defined as a bullied experience by peers in a neighbourhood or school before the participants were 17 years of age.

Abbreviations: SMD, standardized mean difference; BMI, body mass index; SBP, systolic blood pressure; DBP, diastolic blood pressure; HbA1c, glycated hemoglobin A1c.

Table S4. Schoenfeld residuals tests for analyzing childhood exposure to interparental physical violence and verbal conflict with the risk of adult-onset diabetes in model 2

	Chisq statistics*	P value	Global Chisq statistics*	Global P value
**Interparental physical violence**				
Exposed[b] vs. Unexposed[a]	2.429	0.119	18.309	0.306
Frequently, Sometimes vs. Unexposed[a]	3.722	0.156	19.765	0.286
Frequently, Sometimes, Rarely vs. Never	4.457	0.216	20.556	0.302
**Interparental verbal conflict**				
Exposed[b] vs. Unexposed[a]	0.0004	0.983	16.300	0.434
Frequently, Sometimes vs. Unexposed[a]	0.029	0.986	16.370	0.498
Frequently, Sometimes, Rarely vs. Never	0.260	0.967	16.603	0.551

*Chisq statistic for the exposure variable; Global Chisq statistics for the entire model.

[a]Unexposed was defined as witnessing interparental physical violence/verbal conflict never or rarely when participants were younger than 17 years; [c]Exposed was defined as witnessing interparental physical violence/verbal conflict sometimes or frequently when participants were younger than 17 years.

Abbreviations: HR, hazard ratio; CI, confidence interval; Ref, reference.

Model 2: adjusted for sex, age, smoking, drinking, education, rural residence, highest parental education, childhood hunger, childhood bullying experience, and childhood sibling violence.

Table S5. Sensitivity analysis 1: Associations of childhood exposure to interparental physical violence and verbal conflict with the risk of adult-onset diabetes using interval censored Cox regression model

Exposure	Total, N	No. of Events	Crude model		Model 1		Model 2	
		(incidence rate[a])	HR [95% CI]	P value	HR [95% CI]	P value	HR [95% CI]	P value
**Interparental physical violence**								
Exposure to witnessing interparental physical violence								
Unexposed[b]	5,437	667 (19.2)	Ref		Ref		Ref	
Exposed[c]	505	100 (31.9)	1.70 [1.50, 1.89]	<0.001	1.70 [1.50, 1.89]	<0.001	1.67 [1.46, 1.88]	<0.001
**Interparental verbal conflict**								
Exposure to witnessing interparental verbal conflict								
Unexposed[b]	4,626	574 (19.5)	Ref		Ref		Ref	
Exposed[c]	1,316	193 (23.2)	1.21 [1.06, 1.36]	0.013	1.22 [1.06, 1.38]	0.013	1.25 [1.09, 1.42]	0.006

[a]The incidence rate was expressed per 1,000 person-years; [b]Unexposed was defined as witnessing interparental physical violence/verbal conflict never or rarely when participants were younger than 17 years; [c]Exposed was defined as witnessing interparental physical violence/verbal conflict sometimes or frequently when participants were younger than 17 years.

Abbreviations: HR, hazard ratio; CI, confidence interval; Ref, reference.

Model 1: adjusted for sex, and age.

Model 2: Model 1+ further adjusted for smoking, drinking, education, rural residence, highest parental education, childhood hunger, childhood bullying experience, and childhood sibling violence.

Table S6. Baseline characteristics of study population in 1:1 propensity score matched cohorts

	Exposure to witnessing interparental physical violence			Exposure to witnessing interparental verbal conflict		
Variable	Unexposed (n=493)	Exposed (n=493)	SMD	Unexposed (n=1,399)	Exposed (n=1,399)	SMD
Age, years	58.7 (9.0)	58.7 (8.7)	0.005	58.3 (8.8)	58.0 (8.7)	0.037
Male, n (%)	226 (45.8)	222 (45.0)	0.016	626 (44.7)	648 (46.3)	0.032
Education, n (%)			0.046			<0.001
Middle school or below	452 (91.7)	458 (92.9)		1,260 (90.1)	1,260 (90.1)	
High school or above	41 (8.3)	35 (7.1)		139 (9.9)	139 (9.9)	
Rural residence, n (%)	338 (68.6)	344 (69.8)	0.026	936 (66.9)	915 (65.4)	0.032
Childhood hunger, n (%)	376 (76.3)	377 (76.5)	0.005	1,067 (76.3)	1,053 (75.3)	0.023
Frequency of childhood bullying experience, n (%)			0.047			0.028
Never	72 (14.6)	80 (16.2)		896 (64.0)	907 (64.8)	
Rarely	67 (13.6)	64 (13.0)		239 (17.1)	240 (17.2)	
Sometimes	27 (5.5)	26 (5.3)		186 (13.3)	182 (13.0)	
Frequently	327 (66.3)	323 (65.5)		78 (5.6)	70 (5.0)	0.003
Frequency of childhood sibling violence, n (%)			0.080			0.054
Never	412 (83.6)	409 (83.0)		1,187 (84.8)	1,167 (83.4)	
Rarely	49 (9.9)	54 (11.0)		140 (10.0)	148 (10.6)	
Sometimes	27 (5.5)	28 (5.7)		65 (4.6)	72 (5.1)	
Frequently	5 (1.0)	2 (0.4)		7 (0.5)	12 (0.9)	
Highest parental education, n (%)			0.047			0.017
Illiterate	279 (56.6)	290 (58.8)		791 (56.5)	803 (57.4)	
Middle school or below	197 (40.0)	188 (38.1)		563 (40.2)	552 (39.5)	
High school or above	17 (3.4)	15 (3.0)		45 (3.2)	44 (3.1)	
Smoking, n (%)	153 (31.0)	153 (31.0)	<0.001	402 (28.7)	411 (29.4)	0.014
Drinking, n (%)	174 (35.3)	174 (35.3)	<0.001	502 (35.9)	501 (35.8)	0.001

Data were expressed as mean (SD), median [25th percentile, 75th percentile] or as frequencies (percentages).

Unexposed was defined as witnessing interparental physical violence/verbal conflict never or rarely when participants were younger than 17 years; Exposed was defined as witnessing interparental physical violence/verbal conflict sometimes or frequently when participants were younger than 17 years.

Childhood bullying experience was defined as a bullied experience by peers in a neighbourhood or school before the participants were 17 years of age.

Abbreviations: SMD, standardized mean difference.

Table S7. Sensitivity analysis 2: Associations of childhood exposure to interparental physical violence and verbal conflict with the risk of adult-onset diabetes in 1:1 propensity score matched cohorts

Exposure	Total, N	No. of Events (incidence rate[a])	Crude model		Model 1		Model 2	
			HR [95% CI]	P value	HR [95% CI]	P value	HR [95% CI]	P value
**Interparental physical violence**								
Exposure to witnessing interparental physical violence								
Unexposed[b]	493	61 (19.6)	Ref		Ref		Ref	
Exposed[c]	493	86 (28.3)	1.44 [1.04, 2.01]	0.028	1.45 [1.04, 2.01]	0.027	1.42 [1.02, 1.97]	0.038
**Interparental verbal conflict**								
Exposure to witnessing interparental verbal conflict								
Unexposed[b]	1,399	173 (19.4)	Ref		Ref		Ref	
Exposed[c]	1,399	202 (22.8)	1.18 [0.99, 1.43]	0.085	1.18 [0.99, 1.45]	0.087	1.19 [0.99, 1.44]	0.086

[a]The incidence rate was expressed per 1,000 person-years; [b]Unexposed was defined as witnessing interparental physical violence/verbal conflict never or rarely when participants were younger than 17 years; [c]Exposed was defined as witnessing interparental physical violence/verbal conflict sometimes or frequently when participants were younger than 17 years.

Abbreviations: HR, hazard ratio; CI, confidence interval; Ref, reference.

Model 1: adjusted for sex, and age.

Model 2: Model 1+ further adjusted for smoking, drinking, education, rural residence, highest parental education, childhood hunger, childhood bullying experience, and childhood sibling violence.

Table S8. Sensitivity analysis 3: Associations of childhood exposure to interparental physical violence and verbal conflict with the risk of adult-onset diabetes using inverse probability of treatment weighting and overlap weighting

Exposure	Total, N	No. of Events (incidence rate[a])	Crude model		Model 1		Model 2	
			HR [95% CI]	P value	HR [95% CI]	P value	HR [95% CI]	P value
**Inverse probability of treatment weighting**								
**Interparental physical violence**								
Exposure to witnessing interparental physical violence								
Unexposed[b]	6,204	744 (18.8)	Ref		Ref		Ref	
Exposed[c]	565	110 (31.5)	1.62 [1.30, 2.01]	<0.001	1.62 [1.31, 2.01]	<0.001	1.64 [1.32, 2.04]	<0.001
**Interparental verbal conflict**								
Exposure to witnessing interparental verbal conflict								
Unexposed[b]	5,246	634 (19.0)	Ref		Ref		Ref	
Exposed[c]	1,523	220 (22.9)	1.18 [1.01, 1.39]	0.037	1.18 [1.01, 1.39]	0.037	1.19 [1.01, 1.39]	0.034
**Overlap weighting**								
**Interparental physical violence**								
Exposure to witnessing interparental physical violence								
Unexposed[b]	6,204	744 (18.8)	Ref		Ref		Ref	
Exposed[c]	565	110 (31.5)	1.80 [1.43, 2.26]	<0.001	1.82 [1.45, 2.28]	<0.001	1.81 [1.45, 2.26]	<0.001
**Interparental verbal conflict**								
Unexposed[b]	5,246	634 (19.0)	Ref		Ref		Ref	
Exposed[c]	1,523	220 (22.9)	1.21 [1.03, 1.42]	0.023	1.22 [1.04, 1.44]	0.014	1.21 [1.03, 1.43]	0.024

[a]The incidence rate was expressed per 1,000 person-years; [b]Unexposed was defined as witnessing interparental physical violence/verbal conflict never or rarely when participants were younger than 17 years; [c]Exposed was defined as witnessing interparental physical violence/verbal conflict sometimes or frequently when participants were younger than 17 years.

Abbreviations: HR, hazard ratio; CI, confidence interval; Ref, reference.

Model 1: adjusted for sex, and age.

Model 2: Model 1+ further adjusted for smoking, drinking, education, rural residence, highest parental education, childhood hunger, childhood bullying experience, and childhood sibling violence.

Table S9. Sensitivity analysis 4: Associations of childhood exposure to interparental physical violence and verbal conflict with the risk of adult-onset diabetes after accounting for missing data using inverse probability of treatment weighting and overlap weighting

Exposure	Total, N	No. of Events	**Crude model**		**Model 1**		**Model 2**	
		(incidence rate[a])	HR [95% CI]	P value	HR [95% CI]	P value	HR [95% CI]	P value
**Inverse probability of treatment weighting**								
**Interparental physical violence**								
Exposure to witnessing interparental physical violence								
Unexposed[b]	6,204	744 (18.8)	Ref		Ref		Ref	
Exposed[c]	565	110 (31.5)	2.00 [1.57, 2.54]	<0.001	1.99 [1.57, 2.54]	<0.001	2.00 [1.57, 2.55]	<0.001
**Interparental verbal conflict**								
Exposure to witnessing interparental verbal conflict								
Unexposed[b]	5,246	634 (19.0)	Ref		Ref		Ref	
Exposed[c]	1,523	220 (22.9)	1.22 [1.00, 1.47]	0.045	1.22 [1.00, 1.47]	0.046	1.21 [1.00, 1.47]	0.048
**Overlap weighting**								
**Interparental physical violence**								
Exposure to witnessing interparental physical violence								
Unexposed[b]	6,204	744 (18.8)	Ref		Ref		Ref	
Exposed[c]	565	110 (31.5)	1.99 [1.56, 2.52]	<0.001	1.99 [1.57, 2.52]	<0.001	1.99 [1.56, 2.52]	<0.001
**Interparental verbal conflict**								
Unexposed[b]	5,246	634 (19.0)	Ref		Ref		Ref	
Exposed[c]	1,523	220 (22.9)	1.23 [1.01, 1.48]	0.036	1.23 [1.01, 1.48]	0.035	1.22 [1.00, 1.48]	0.045

[a]The incidence rate was expressed per 1,000 person-years; [b]Unexposed was defined as witnessing interparental physical violence/verbal conflict never or rarely when participants were younger than 17 years; [c]Exposed was defined as witnessing interparental physical violence/verbal conflict sometimes or frequently when participants were younger than 17 years.

Abbreviations: HR, hazard ratio; CI, confidence interval; Ref, reference.

Model 1: adjusted for sex, and age.

Model 2: Model 1+ further adjusted for smoking, drinking, education, rural residence, highest parental education, childhood hunger, childhood bullying experience, and childhood sibling violence.

Table S10. Sensitivity analysis 5: Associations of childhood exposure to interparental physical violence and verbal conflict with the risk of adult-onset diabetes using a modified exposure definition

Exposure	Total, N	No. of Events	**Crude model**		**Model 1**		**Model 2**	
		(incidence rate[a])	HR [95% CI]	P value	HR [95% CI]	P value	HR [95% CI]	P value
**Interparental physical violence**								
Exposure to witnessing interparental physical violence								
Unexposed[b]	5,345	632 (18.5)	Ref		Ref		Ref	
Exposed[c]	1,424	222 (24.8)	1.33 [1.14, 1.55]	<0.001	1.34 [1.15, 1.57]	<0.001	1.31 [1.12, 1.54]	0.001
Exposure levels of witnessing interparental physical violence								
Unexposed[b]	5,345	632 (18.5)	Ref		Ref		Ref	
Rarely	859	112 (20.5)	1.08 [0.89, 1.33]	0.426	1.10 [0.90, 1.35]	0.333	1.08 [0.88, 1.32]	0.486
Sometimes to Frequently	565	110 (31.5)	1.72 [1.40, 2.10]	<0.001	1.72 [1.41, 2.11]	<0.001	1.69 [1.38, 2.08]	<0.001
**Interparental verbal conflict**								
Exposure to witnessing interparental verbal conflict								
Unexposed[b]	5,345	632 (18.5)	Ref		Ref		Ref	
Exposed[c]	3,514	467 (20.9)	1.11 [0.97, 1.28]	0.114	1.16 [1.01, 1.32]	0.036	1.15 [1.01, 1.32]	0.042
Exposure levels of witnessing interparental verbal conflict								
Unexposed[b]	5,345	632 (18.5)	Ref		Ref		Ref	
Rarely	1,991	247 (19.4)	1.04 [0.88, 1.22]	0.659	1.08 [0.92, 1.27]	0.336	1.07 [0.91, 1.27]	0.390
Sometimes to Frequently	1,523	220 (22.9)	1.22 [1.03, 1.44]	0.019	1.25 [1.06, 1.48]	0.008	1.23 [1.04, 1.46]	0.017

[a]The incidence rate was expressed per 1,000 person-years; [b]Unexposed was defined as witnessing interparental physical violence/verbal conflict never when participants were younger than 17 years; [c]Exposed was defined as witnessing interparental physical violence/verbal conflict rarely, sometimes or frequently when participants were younger than 17 years.

Abbreviations: HR, hazard ratio; CI, confidence interval; Ref, reference.

Model 1: adjusted for sex, and age.

Model 2: Model 1+ further adjusted for smoking, drinking, education, rural residence, highest parental education, childhood hunger, childhood bullying experience, and childhood sibling violence.

Table S11. Sensitivity analysis 6: Associations of childhood exposure to interparental physical violence and verbal conflict with the risk of adult-onset diabetes after excluding participants who developed diabetes in 2013

Exposure	Total, N	No. of Events (incidence rate[a])	**Crude model**		**Model 1**		**Model 2**	
			HR [95% CI]	P value	HR [95% CI]	P value	HR [95% CI]	P value
**Interparental physical violence**								
Exposure to witnessing interparental physical violence								
Unexposed[b]	5,997	650 (16.6)	Ref		Ref		Ref	
Exposed[c]	540	101 (29.3)	1.79 [1.45, 2.21]	<0.001	1.80 [1.46, 2.22]	<0.001	1.76 [1.42, 2.18]	<0.001
**Interparental verbal conflict**								
Exposure to witnessing interparental verbal conflict								
Unexposed[b]	5,069	559 (16.9)	Ref		Ref		Ref	
Exposed[c]	1,468	192 (20.2)	1.19 [1.01, 1.40]	0.038	1.20 [1.02, 1.42]	0.028	1.18 [1.00, 1.40]	0.048

[a]The incidence rate was expressed per 1,000 person-years; [b]Unexposed was defined as witnessing interparental physical violence/verbal conflict never or rarely when participants were younger than 17 years; [c]Exposed was defined as witnessing interparental physical violence/verbal conflict sometimes or frequently when participants were younger than 17 years.

Abbreviations: HR, hazard ratio; CI, confidence interval; Ref, reference.

Model 1: adjusted for sex, and age.

Model 2: Model 1+ further adjusted for smoking, drinking, education, rural residence, highest parental education, childhood hunger, childhood bullying experience, and childhood sibling violence.

Table S12. Sensitivity analysis 7: Associations of childhood exposure to interparental physical violence and verbal conflict with the risk of adult-onset diabetes using self-reported diabetes only

Exposure	Total, N	No. of Events (incidence rate[a])	Crude model		Model 1		Model 2	
			HR [95% CI]	P value	HR [95% CI]	P value	HR [95% CI]	P value
**Interparental physical violence**								
Exposure to witnessing interparental physical violence								
Unexposed[b]	6,204	477 (11.8)	Ref		Ref		Ref	
Exposed[c]	565	81 (22.5)	1.96 [1.55, 2.48]	<0.001	1.96 [1.55, 2.48]	<0.001	1.96 [1.54, 2.49]	<0.001
**Interparental verbal conflict**								
Exposure to witnessing interparental verbal conflict								
Unexposed[b]	5,246	411 (12.0)	Ref		Ref		Ref	
Exposed[c]	1,523	147 (14.9)	1.23 [1.02, 1.49]	0.028	1.24 [1.02, 1.49]	0.027	1.23 [1.01, 1.49]	0.038

[a]The incidence rate was expressed per 1,000 person-years; [b]Unexposed was defined as witnessing interparental physical violence/verbal conflict never or rarely when participants were younger than 17 years; [c]Exposed was defined as witnessing interparental physical violence/verbal conflict sometimes or frequently when participants were younger than 17 years.

Abbreviations: HR, hazard ratio; CI, confidence interval; Ref, reference.

Model 1: adjusted for sex, and age.

Model 2: Model 1+ further adjusted for smoking, drinking, education, rural residence, highest parental education, childhood hunger, childhood bullying experience, and childhood sibling violence.
